# Homorepeat variability within the human population

**DOI:** 10.1093/nargab/lqae053

**Published:** 2024-05-20

**Authors:** Pablo Mier, Miguel A Andrade-Navarro, Enrique Morett

**Affiliations:** Institute of Organismic and Molecular Evolution, Faculty of Biology, Johannes Gutenberg University Mainz, Hanns-Dieter-Hüsch-Weg 15, 55128 Mainz, Germany; Institute of Organismic and Molecular Evolution, Faculty of Biology, Johannes Gutenberg University Mainz, Hanns-Dieter-Hüsch-Weg 15, 55128 Mainz, Germany; Departamento de Ingeniería Celular y Biocatálisis, Instituto de Biotecnología, Universidad Nacional Autónoma de México (UNAM), Av. Universidad 2001, Cuernavaca, Morelos 62210, Mexico

## Abstract

Genetic variation within populations plays a crucial role in driving evolution. Unlike the average protein sequence, the evolution of homorepeats can be influenced by DNA replication slippage, when DNA polymerases either add or skip repeats of nucleotides. While there are some diseases known to be caused by abnormal changes in the length of amino acid homorepeats, naturally occurring variations in homorepeat length remain relatively unexplored. In our study, we examined the variation in amino acid homorepeat length of human individuals by analyzing 125 748 exomes, as well as 15 708 whole genomes. Our analyses revealed significant variability in homorepeat length across the human population, indicating that these motifs are prone to mutations at higher rates than non repeat sequences. We focused our study on glutamine homorepeats, also known as polyQ sequences, and found that shorter polyQ sequences tend to exhibit greater length variation, while longer ones primarily undergo deletions. Notably, polyQ sequencesthat are more conserved across primates tend to show less variation within the human population, indicating stronger selective pressure to maintain their length. Overall, our results demonstrate that there is large natural variation in the length of homorepeats within the human population, with no apparent impact on observable traits.

## Introduction

Homorepeats (polyX), contiguous tracts of a repeated amino acid, accumulate in particular protein families (1), mostly within intrinsically disordered regions (2). Their length changes along evolution indicating that they have length-dependent functionality (3). They have functions related to the modulation of protein interactions (4), and some homorepeat expansions may lead to the formation of aberrant pathological interactions (5).

Consequently, alterations in the length of certain homorepeats have severe impacts on health, and many diseases are a direct result of the extension of homorepeats in critical proteins (6,7). For example, the expansion of polyA has been linked to nine developmental diseases (8), while the expansion of polyQ results in nine neurodegenerative diseases where the length of the resulting polyQ correlates with the onset and severity of the condition (9,10).

Because of this, homorepeats appear in proteins stringently regulated (4), and evolutionary studies show that homorepeats undergo a delicate balance where DNA slippage produces homorepeat length variation (11) but selection mechanisms introduce synonymous mutations to counteract this variation (12). Constrains on homorepeat variation have been studied across species (13), however, it is not clear how resilient the human species is to homorepeat length variation. Population data offers a resource to investigate this question.

The study of genetic polymorphisms in a population is a means of detecting neutral variations or variants in specific populations that result from selection for a particular environment (14). The larger the number of individuals studied, the more information can be gathered and therefore more precise accounts of the phenotypic effect of variation can be obtained. Alleles that widely vary tend to be of little phenotypic consequence. In contrast, conserved alleles imply strong functional constraints (15).

The human species has very limited genetic variability compared to other species, including our closest living relatives (16). However, the large population size of our species and the everyday increasing volume of genetic data for millions of individuals result in a large number of alternative alleles (15,17,18). The rapid human population growth resulted in an excess of rare variants (19). Since the large majority of the individuals sequenced are healthy adults or with very specific health issues impacting only a few alleles, the data gathered is of immense value to understand genetic variation with no strong functional consequences.

In this work, we characterized the variation of amino acid homorepeats in the human proteome at the population level using public allele data covering more than one hundred and forty thousand individuals (the Genome Aggregation Database (20). Our results show that homorepeats are more variable than random regions of similar length, and that the variation is directly related to the length of the homorepeat, therefore they can be seen as mutational hotspots. PolyQ stood out as the most variable homorepeat. Large polyQ varied mainly in size with few missense variations, while shorter polyQ showed a large proportion of different missense variants with little size variation. Evolutionarily conserved polyQ motifs were more conserved within the human population, implying stronger functional constraints. Our work highlights the variability of homorepeat length and composition in humans.

## Materials and methods

We downloaded a set of chromosome variation sites obtained from human exomes (with data from 125 748 exomes), and the sites from exome calling intervals from human genomes (with data from 15 708 whole genomes), from the Genome Aggregation Database (gnomAD) v2.1.1 database (20). There is a newer version of the database (v3), but it currently lacks exome information. We selected variants within protein-coding regions and associated them with an Ensembl protein ID for downstream analysis. Variants resulting in synonymous codons were not considered. We simplified the information available to describe each variant only by protein ID, amino acid in the reference human proteome, amino acid sequence position, alternative amino acid or modification (duplication, deletion, stop codon or frameshift), and number of reported individuals with the variant (allele count, AC). We produced three independent sets of variants, including (i) variants obtained from exomes and present in at least 100 individuals (AC ≥ 100), (ii) variants obtained from genomes and present in at least 100 individuals and (iii) variants obtained both from exomes and genomes and present in at least 10 individuals.

We downloaded the complete human reference proteome (UP000005640) from UniProtKB release 2023_02 (21), and to avoid redundancy we limited it to one protein sequence per gene, as provided in UniProtKB (20 593 proteins). We mapped these proteins to the variants through the Ensembl protein ID with the mapping tool ‘ID mapping’ provided by UniProtKB.

The orthology relationships between human protein coding sequences and proteins from six different primates were retrieved from Ensembl/Biomart release 109 (22). The species used were: Pan troglodytes (chimpanzee, Pan_tro_3.0), *Gorilla gorilla gorilla* (gorilla, gorGor4), *Pongo abelii* (Sumatran orangutan, Susie_PABv2), *Chlorocebus sabaeus* (green monkey, ChlSab1.1), *Papio anubis* (olive baboon, Panubis1.0) and *Macaca mulatta* (macaque, Mmul_10). The protein sequences from the orthologs were also obtained from the same database.

The sets of orthologs in which the human protein had at least one polyQ region with a minimum of eight glutamines were aligned with MAFFT v7.490 (23).

Although standard deviation assumes that the distributions are normal, we have used it descriptively to compare distributions because it is a simple and intuitive measure and there are not very good alternatives for the type of data analyses presented here.

## Results and discussion

### Longer homorepeats overlap with more variants in the human population

We downloaded information about human genomic polymorphisms from the Genome Aggregation Database (gnomAD (20)), with data from 125 748 exomes and 15 708 whole genomes. We identified variants defined here as a position in a protein-coding sequence plus the type of variation with respect to the reference and the reported allele count (see Materials and methods for details). We considered the sites identified in at least 10 different individuals in both the exomic and genomic datasets (results were comparable when considering either genomic or exomic information independently and considering variants found in at least 100 individuals; data not shown). We identified 164 981 variants, covering 1.45% amino acid positions in the human reference proteome. The amino acid frequencies of the variants are similar to the background of the proteome (Supplementary Figure S1), except for leucine, phenylalanine, and lysine, underrepresented in the variants, and arginine, three-fold more prevalent in them than in the background.

**Figure 1.**
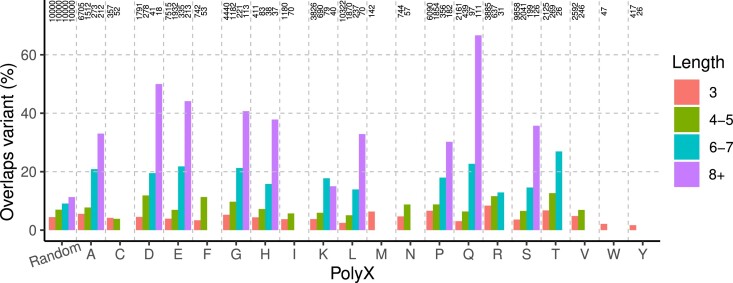
Frequency of overlap to variants for homorepeats with different length ranges. On top, number of homorepeats in the human reference proteome. We used sets of 10000 random regions per length group to establish a baseline.

We calculated pure homorepeats of three or more identical amino acids in the human reference proteome (using polyX2 (24)). We obtained 81 732 homorepeats from 17 007 different proteins. A 5.83% of all homorepeats overlap with at least one variant (Figure 1), with values 4.52%, 7.59%, 18.86% and 38,47% for homorepeats of length 3, 4–5, 6–7 and >8 amino acids, respectively.

Larger sequence regions can be expected to overlap more often with variants just by chance. To evaluate whether the frequency of overlap to variants for each homorepeat length range is significantly large, we calculated overlaps for sets of regions with the same length distribution of each range. While the result for homorepeats of length three is comparable to the one calculated for 10000 random regions of similar length (4.44%), longer homorepeats vary 3.4-fold more than expected (11.29%). PolyQ, polyD, polyE and polyG showed the largest frequency of overlap (>40%). In particular, long polyQ regions stand out with 66.67% variation in the human population. To the best of our knowledge, variation in these regions has only been reported for pathological stretches.

Therefore, our results show that long polyQ regions are mutational hotspots. To follow up this finding, we investigated the types of variants within polyQ (polyQ variants) with different lengths and whether these relate to its evolutionary conservation. Interestingly, we observe that the types of variants depend on the length of the polyQ (Table 1). In polyQ of length 3 (which has a frequency of variants similar to the background), the most frequent variants are missense mutations. On the contrary, the most frequent variants in polyQ of length 4–7 are duplications followed by deletions; that is, variants that change its length, with a bias towards longer regions. Variants changing length are predominant for polyQ of length 8 and above, with deletions and duplications having similar frequencies, although deletions are more than three times more prevalent in the population. The increased length variability of longer polyQ in the human population agrees with the larger variability of longer polyQ seen in primates (25).

**Table 1.** Type and frequency of variant types per polyQ length

PolyQ length	Variant type	Number of sites in polyQ	Sum allele count
3	Gln > Arg	14	105 904
	Gln > His	13	176 822
	Gln > Glu	10	341 790
	Gln > Lys	7	119 149
	Deletion	5	132 770
	Frameshift	3	86 716
	Gln > Pro	2	37 006
	STOP	2	3115
	Duplication	2	262 471
	Gln > Leu	1	637
	Gln > Lys	1	54
4–5	Duplication	10	288 488
	Deletion	5	31 359
	Gln > Arg	4	285 752
	Gln > Pro	4	12 622
	Gln > His	3	544
	Gln > Lys	2	76 022
	Gln > Glu	2	17 314
	Frameshift	1	88
6–7	Duplication	19	76 849
	Deletion	9	166 023
	Frameshift	2	17 524
	Gln > Pro	1	66
	Gln > His	1	241
	Gln > Glu	1	1128
	Gln > Arg	1	754
8+	Deletion	90	1 547 410
	Duplication	81	452 267
	Frameshift	51	579 171
	Gln > Pro	15	30 739
	Gln > His	12	17 591
	Insertion	11	6543
	Gln > Arg	2	1717
	Gln > Glu	2	482

### Highly conserved polyQ in the human population are more evolutionarily stable

**Figure 2.**
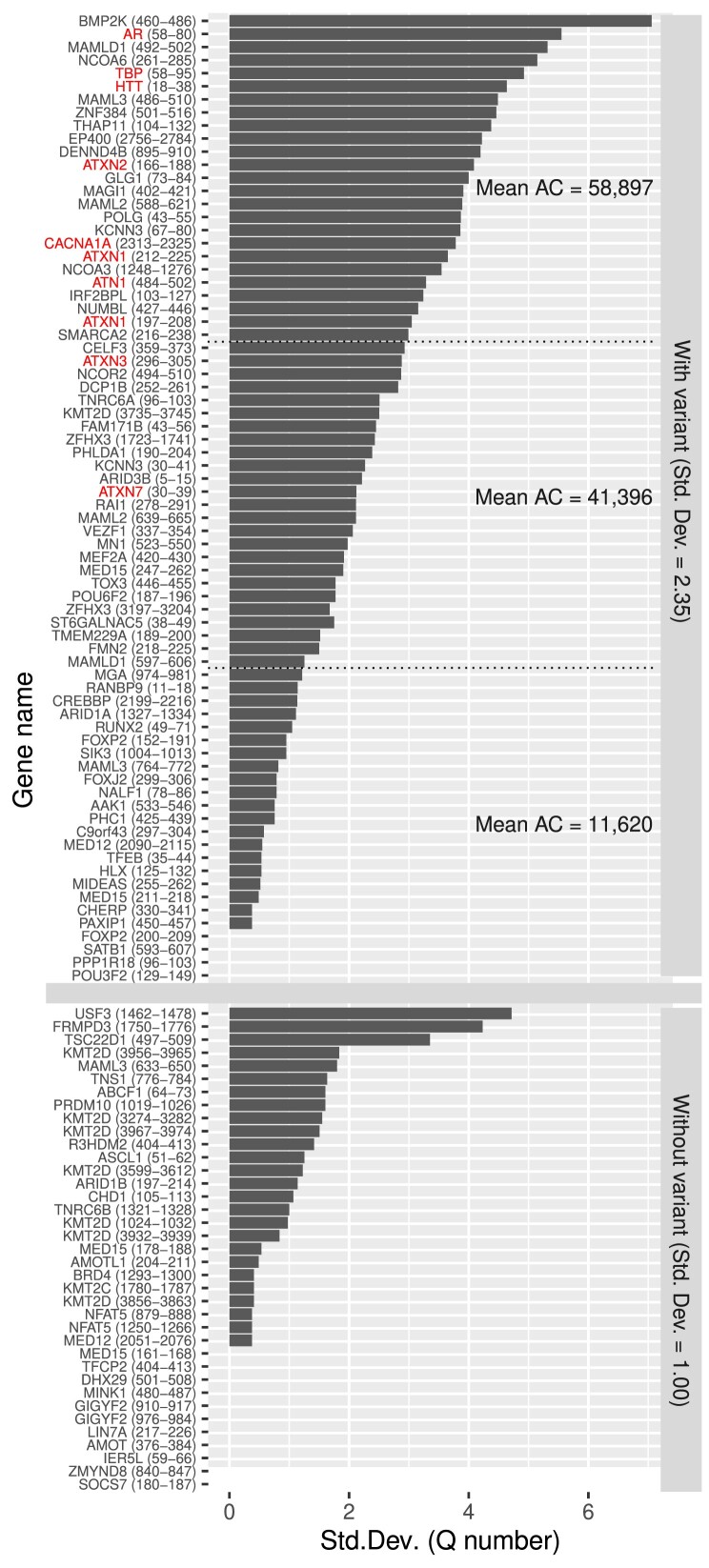
PolyQ variability in primates for long human polyQ. Each human protein with a long polyQ (length ≥ 8 amino acids) was aligned to their orthologs in six primates (multiple sequence alignment, see Methods for details). For each human long polyQ (a protein can have multiple ones), the number of glutamines in the aligned sequence of each primate ortholog was counted. The bars represent the standard deviation of those six values (or fewer if orthologs were missing). Top: human polyQ with variants. Bottom: human polyQ without variants. PolyQ associated with human pathologies are highlighted in red. The polyQ variants were divided in three groups with high, medium and low polyQ conservation in primates for analysis (see text for details).

We next addressed the question whether variable polyQ in humans are also variable in primates. We obtained the orthologs from the 90 human proteins containing the set of 111 long (length ≥ 8) polyQ regions, in 6 different primates with various speciation times (according to TimeTree (26)): 6.4 myr (*Pan troglodytes*), 8.6 myr (*Gorilla gorilla*), 15.2 myr (*Pongo abelii*) and 28.8 myr (*Chlorocebus sabaeus*, *Papio anubis* and *Macaca mulatta*). We aligned the sets of orthologs and counted the number of glutamine residues aligning with the human reference polyQ regions. We then computed the standard deviation of the number of glutamines per polyQ region in each set of orthologs (Supplementary File S2). We observed that polyQ that overlap human variants have more variation in primates compared to those that have no variants, with a mean standard deviation in the number of aligned glutamines of 2.35 and 1.00, respectively (Figure 2).

We then explored how the degree of polyQ variation in humans relates to their evolutionary conservation. To do this, we split the human polyQ variants into three groups based on the extent of variation seen in primates: large, medium, and low (Figure 2). On average, the number of variants per polyQ region were 58 897, 41 396 and 11 620 respectively (Supplementary File S1). However, it is important to note that longer polyQ sequences naturally have a higher likelihood of accumulating variations and exhibiting greater length divergence among primates. Accordingly, the average length of polyQ sequences across primates in each group was 21.2, 13.4 and 13 amino acids respectively. Human polyQ sequences without variants were even shorter on average, at 11 amino acids. To ensure our analysis is not biased by sequence length, we compared sets of polyQ sequences of the same length, with and without variants. For instance, among polyQ sequences of length eight, the 13 with variants have an average standard deviation of 0.96, whereas the 14 without variants averaged only 0.48. Similarly, among polyQ sequences of length 10, the 9 sequences with variants averaged a standard deviation of 1.57, while the 6 without variants averaged 0.87. Although the influence of sequence length cannot be completely eliminated, we observed that when comparing groups of human polyQ sequences with or without variants, those with variants exhibited greater variation compared to other non-human primate species.

Finally, most of the polyQ regions involved in disease (8 out of 10) are in the group with the greatest variability in polyQ length in primates (Figure 2). In this group of 25 proteins, disease-associated polyQ regions have a comparable average standard deviation of the number of glutamines to non-disease associated ones (4.12 and 4.22, respectively), but a 2.4-fold depletion in the allele count (29 943 and 72 523 alleles on average, respectively) (Supplementary File S2).

## Conclusions

We have carried out the first study of homorepeat variation using large-scale population data. Homorepeats have different properties depending on their length and this is also the case for their natural variation. The observation that homorepeats of length three are not particularly different than random in their accumulation of variants, suggests that they might be too short to have special functional or mutational properties, making them subject to evolutionary pressure similar to that of non-repeated sequences. This result agrees with the observation that polyQ regions of length four and above have a particular structural context, while polyQ of length three do not (27).

When focusing on longer homorepeats (of length ≥ 8), polyQ is the one that accumulates more variants (>60%), higher than random regions of similar length (around 10%). For these longer polyQ, variants changing the homorepeat length are dominant, with deletions and duplications similarly frequent, reflecting gene slippage acting to change the number of repeats. In contrast, polyQ of intermediate length (4–7) have duplications twice as frequently as deletions. Homorepeat length changing variants are almost absent for polyQ of length 3, again suggesting that they behave as non-repeated sequences. Given the many examples of pathogenicity of polyQ expansion, we speculate that the long polyQ have reached an equilibrium of optimal length, while polyQ of intermediate length do not have evolutionary pressure to reduce their size and have room to expand.

Comparison with non-human primate orthologs showed that polyQ with variation in humans are also less evolutionarily conserved. While there is an effect from polyQ size, where longer polyQ tend to be more variable in any comparison, when restricting our analysis to polyQ of only one size we could also observe the correlation between larger variation in human population and in evolution. Together, our observations indicate that homorepeat variation in the population is frequent, suggesting that gene slippage is acting as an evolutionary process. Our results support the idea that when a homorepeat reaches a size greater than three, it enters a regime where it could expand further, and that population variation reflects evolutionary variation and thus both reflect similarly functional constraints in the sequence. These observations have been made possible by the availability of massive amounts of population data and promise that the future expansion of such resources can be used to increase our understanding of human biology and evolution.

## Availability and implementation

The datasets used and/or analyzed during the current study are available from https://cbdm-01.zdv.uni-mainz.de/∼munoz/polyx_population/.

## Supplementary data

Supplementary Data are available at NARGAB Online.

## Supplementary Material

lqae053_Supplemental_Files
