# Supplementary material for: Homorepeat variability within the human population
Source: NAR Genom Bioinform. 2024 May 20;6(2):lqae053. doi: 10.1093/nargab/lqae053 (PMC11106027; doi:10.1093/nargab/lqae053)
Supplement: lqae053_Supplemental_Files [file lqae053_supplemental_files.zip › Supplementary_Materials.docx]

# **Supplementary Materials**

**Supplementary File 1.** PolyX regions identified in the human reference proteome and overlap with variants.

**Supplementary File 2.** Number of glutamines per ortholog aligned with the human polyQ region, for long human polyQ regions with and without variants; in red, the human polyQ regions associated with disease.


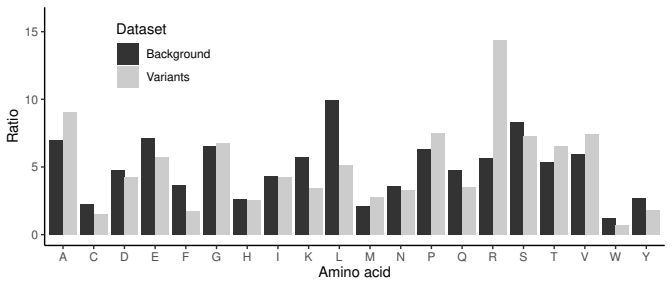


**Supplementary Figure 1.** Amino acid usage of the human reference proteome and variants.
